# Caspase-2 deficiency enhances whole-body carbohydrate utilisation and prevents high-fat diet-induced obesity

**DOI:** 10.1038/cddis.2017.518

**Published:** 2017-10-26

**Authors:** Claire H Wilson, Andrej Nikolic, Stephen J Kentish, Marianne Keller, George Hatzinikolas, Loretta Dorstyn, Amanda J Page, Sharad Kumar

**Affiliations:** 1 Centre for Cancer Biology, University of South Australia and SA Pathology, Adelaide, South Australia, Australia; 2 Adelaide Medical School, University of Adelaide, Adelaide, South Australia, Australia; 3 South Australian Health and Medical Research Institute (SAHMRI), Adelaide, South Australia, Australia

## Abstract

Caspase-2 has been shown to be involved in metabolic homeostasis. Here, we show that caspase-2 deficiency alters basal energy metabolism by shifting the balance in fuel choice from fatty acid to carbohydrate usage. At 4 weeks of age, whole-body carbohydrate utilisation was increased in *Casp2*^−/−^ mice and was maintained into adulthood. By 17 weeks of age, *Casp2*^−/−^ mice had reduced white adipose mass, smaller white adipocytes decreased fasting blood glucose and plasma triglycerides but maintained normal insulin levels. When placed on a 12-week high-fat diet (HFD), *Casp2*^−/−^ mice resisted the development of obesity, fatty liver, hyperinsulinemia and insulin resistance. In addition, HFD-fed *Casp2*^−/−^ mice had reduced white adipocyte hypertrophy, apoptosis and expansion of both subcutaneous and visceral adipose depots. Increased expression of *UCP1* and the maintenance of *adiponectin* levels in white adipose tissue of HFD-fed *Casp2*^−/−^ mice indicated increased browning and adipocyte hyperplasia. We found that while the preference for whole-body carbohydrate utilisation was maintained, HFD-fed *Casp2*^−/−^ mice were not impaired in their ability to switch to utilising fats as a fuel source. Our findings suggest that caspase-2 impacts basal energy metabolism by regulating adipocyte biology and fat expansion, most likely via a non-apoptotic function. Furthermore, we show that caspase-2 deficiency shifts the balance in fuel choice towards increased carbohydrate utilisation and propose that this is due to mild energy stress. As a consequence, *Casp2*^−/−^ mice show an adaptive remodelling of adipose tissue that protects from HFD-induced obesity and improves glucose homeostasis while paradoxically increasing their susceptibility to oxidative stress induced damage and premature ageing.

Whole-body energy homeostasis is vital for healthy ageing and survival^1, 2^ with its perturbation contributing to the development of numerous disease including obesity, type II diabetes and cancer.^1, 2, 3, 4^ In response to changes in energy supply and demand, fuel choice (fat *versus* carbohydrate), conversion, utilisation and storage fluctuate to maintain energy homeostasis.^1, 5^ This involves multiple levels of complex regulation and cross-talk between different organs, tissues and cell types.^1^ Such fluctuations in fuel choice also occur with differing cell states and as an adaptive response to stress conditions.^5^ As a consequence, these fluctuations can alter fuel choice in distant organs through systemic communication, causing shifts in whole-body energy metabolism.^5^ The molecular components that control changes and decision for fuel choice is unclear and key to understanding the regulation of energy homeostasis and how its perturbation contributes to diseases such as obesity.

Caspase-2, is the most evolutionary conserved member of the mammalian caspase (cysteine-dependent aspartate specific proteases) family, and is an important regulator of metabolism, ageing and tumour suppression (reviewed in Miles *et al.*^6^ and Puccini *et al.*^7^). Previously, we identified caspase-2 as a potential regulator of lipid metabolism and glucose homeostasis.^8, 9^ In mice, caspase-2 deficiency (*Casp2*^−/−^) results in several signs of premature ageing-related traits^10, 11^ and increased susceptibility to oxidative stress-induced damage and induced tumour formation.^12, 13, 14, 15^ Intriguingly caspase-2 deficiency protects from aged-induced glucose intolerance independent of insulin sensitivity.^8, 9^ Aged *Casp2*^−/−^ mice also have altered body composition (reduced fat and muscle mass),^11^ smaller white adipocytes, enhanced fasting-induced lipolysis of white adipose tissue (WAT) and increased fasting-induced autophagy of skeletal muscle and liver.^8^ In other studies, *Casp2*^−/−^ mice were found to be protected from Western diet (45% kJ from fat, water supplemented fructose and glucose) induced obesity, insulin resistance and non-alcoholic fatty liver disease (NAFLD)^16^ and caspase-2 has been linked with the apoptotic progression of NAFLD to more severe non-alcoholic hepatosteatosis.^17, 18^ Caspase-2 deficiency has also been shown to protect from streptozotocin type I diabetes induced bone marrow adiposity.^19^ These data indicate that caspase-2 may have a direct role in adipocyte biology. However, the precise mechanism of caspase-2 function remains unknown and how its deficiency contributes to improved metabolic outcomes while promoting ageing is yet to be determined.

In this study, we carried out metabolic monitoring with indirect calorimetry and high-fat diet (HFD) feeding (60% kJ from fat) to further investigate the *in vivo* role of caspase-2 in metabolism. We show that *caspase-2* deficiency protects from the development of HFD-induced obesity, NAFLD and insulin resistance. Our data indicate that caspase-2 is an important regulator of glucose homeostasis and basal energy metabolism and supports a role for caspase-2 in modulating adipocyte biology and fat expansion.

## Results

### *Caspase-2* deficiency shifts whole-body fuel utilisation towards increased carbohydrate oxidation

**Figure 1:**
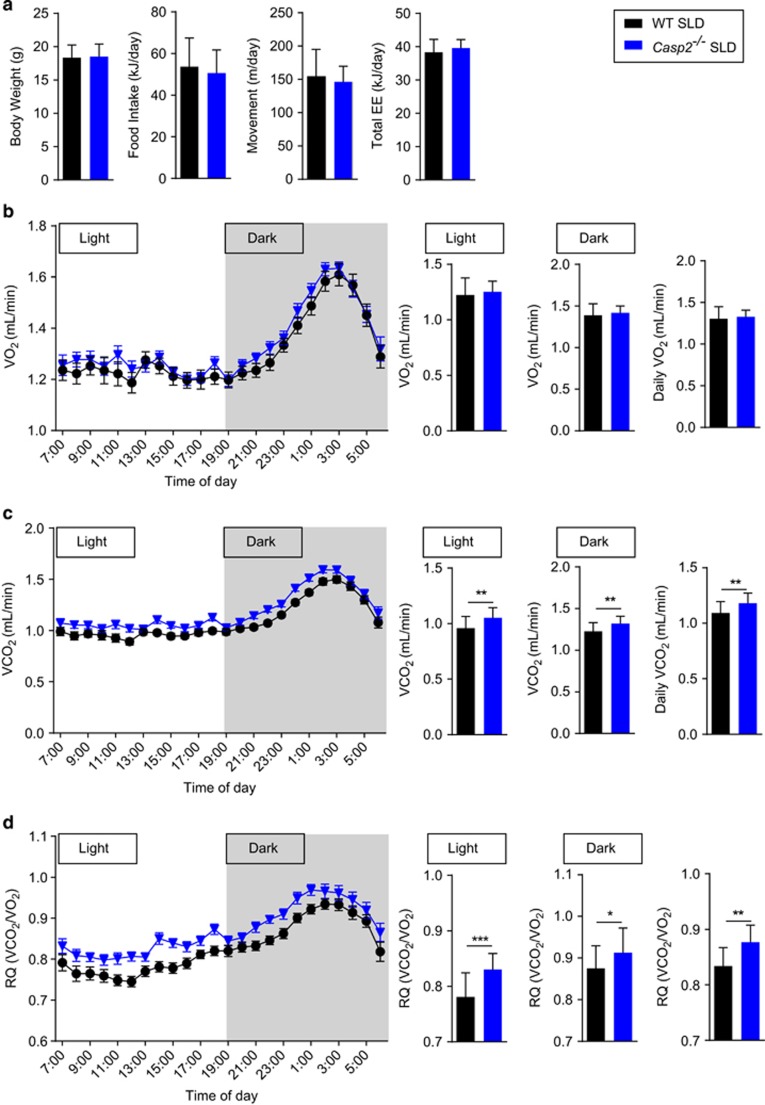
Caspase-2 deficiency shifts whole-body fuel utilisation in SLD-fed mice. Metabolic monitoring with indirect calorimetry performed on 4-week old WT and *Casp2*^−/−^ mice after 1 week of *ad libitum* SLD-feeding. (**a**) Body weight, total daily food intake, activity (movement) and energy expenditure (EE). (**b**) VO_2_ consumption (**c**) VCO_2_ carbon-dioxide production and (**d**) RQ determined over 24- h period with 12-h light–dark cycles. Values are means±S.D. (bar graphs) and means±S.E.M (scatter plots) (*n*=22–23 per group). Statistical significance indicated as *P<0.05, **P<0.01, ***P<0.001

To further investigate the role of caspase-2 in metabolism, we assessed the metabolic phenotype of young *Casp2*^−/−^ mice. At 4 weeks of age (1-week post-weaning) *Casp2*^−/−^ mice and wild-type (WT) controls fed a standard laboratory diet (SLD; 18% kJ from fat) displayed similar body weight, food intake, movement, energy expenditure and feeding behaviour (Figure 1a,Supplementary Figure S1). Indirect calorimetry revealed a significant increase in VCO_2_ production (Figure 1c) in *Casp2*^−/−^ mice but VO_2_ consumption was comparable to WT mice (Figure 1b). Respiratory quotient (RQ), which represents the utilisation of carbohydrate or fat as fuel, was significantly higher in *Casp2*^−/−^ mice (Figure 1d). This indicates that caspase-2 deficiency results in a shift in whole-body fuel utilisation towards increased carbohydrate oxidation.

### *Caspase-2* deficiency protects from HFD-induced obesity, hyperlipidaemia, fatty liver and insulin resistance

**Figure 2:**
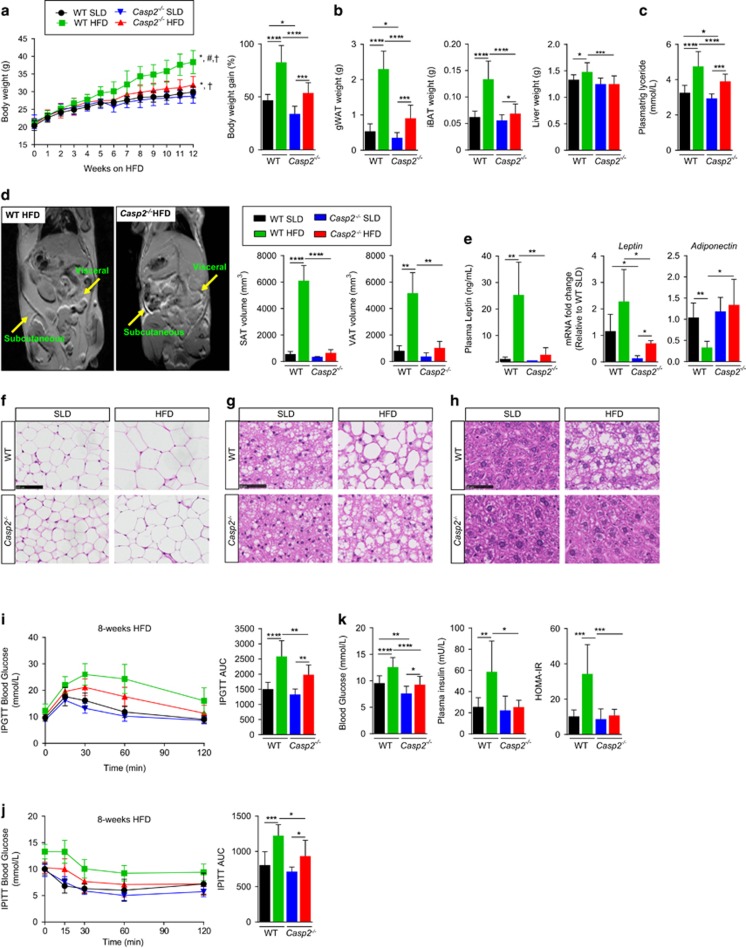
Caspase-2 deficiency protects from HFD-induced obesity and results in metabolic improvements. WT and *Casp2*^−/−^ mice were fed HFD for 12 weeks and blood and tissue collected following a 6-h fast. (**a**) Body weight and weight gain over 12-week period (*n*=11–15 per group). (**b**) Weight of gWAT, iBAT and liver (*n*=14–16). (**c**) Fasting plasma triglycerides (*n*=11–14 per group). (**d**) MRI of HFD-fed mice after 12 weeks and calculated volumes of SAT and VAT (*n*=3 per group). (**e** and **f**) Adipokines assessed by measurement of (**e**) plasma leptin (*n*=8–10), and mRNA expression in gWAT (*n*=5–6). (**f**–**h**) representative images of H&E staining of (**f**) gWAT, (**g**) iBAT and (**h**) liver, scale bar=50 *μ*m. (**i**) IPGTT and (**j**) IPTT was performed on WT and *Casp2*^−/−^ mice (*n*=7–8 per group) at 13 weeks of age after 8 weeks on HFD. (**k**) Fasting blood glucose (*n*=14–16 per group), plasma insulin (*n*=8–10 per group) and calculated homeostatic model assessment of insulin resistance (HOMA-IR) (*n*=8–10 per group) measured after 12 weeks on HFD. Mice were used at 16–17 weeks (including 12 weeks HFD feeding) unless otherwise stated. Values are means±S.D. (bar graphs) or means±S.E.M (scatter plots). Statistical significance indicated as *P<0.05, **P<0.01, ***P<0.001, ****P<0.0001

At 5 weeks of age, mice were either placed on a HFD (60% kJ from fat), or maintained on SLD for 12 weeks. In both dietary groups, *Casp2*^−/−^ mice gained significantly less body weight compared with WT controls and had significantly decreased mass of gonadal adipose tissue (gWAT) (Figures 2a and b). HFD-fed *Casp2*^−/−^ mice also showed reduced mass of interscapular brown adipose tissue (iBAT) and liver compared with HFD-fed WT controls (Figure 2b). Magnetic resonance imaging (MRI) revealed a significant reduction in total body fat content of HFD-fed *Casp2*^−/−^ mice with decreased subcutaneous adipose tissue (SAT) and visceral adipose tissue (VAT) depots (Figure 2d). In both dietary groups, plasma triglycerides were significantly reduced in *Casp2*^−/−^ mice, whereas HFD-fed WT mice developed hypertriglyceridemia (Figure 2c). Levels of plasma cholesterol were not different (data not shown). SLD-fed *Casp2*^−/−^ mice had smaller sized adipocytes in gWAT that were maintained following HFD feeding along with decreased lipid accumulation iBAT (Figures 2f and g). Consistent with differences in fat mass, *Casp2*^−/−^ mice had significantly reduced levels of plasma leptin and *leptin* gene expression in gWAT in both dietary groups and maintained normal *adiponectin* gene expression following HFD feeding (Figure 2e), consistent with previous findings in aged SLD-fed *Casp2*^−/−^mice.^8^

**Figure 3:**
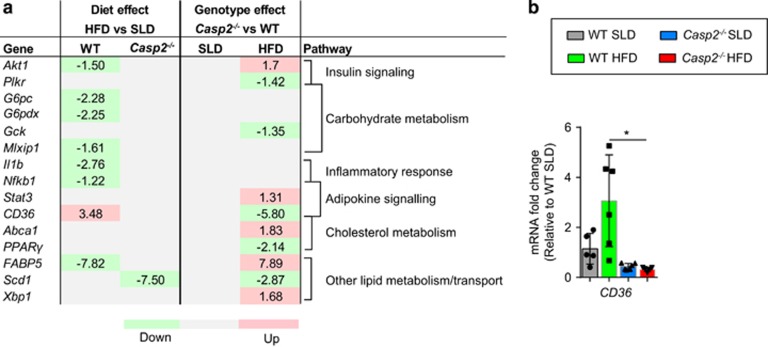
Gene expression analysis of liver tissue from SLD- and HFD-fed WT and *Casp2*^−/−^ mice. Expression of genes known to be involved in fatty liver identified as being significantly altered (fold change relative to SLD-fed WT mice >1.2 and *P*<0.05) by diet or genotype as measured by (**a**) 84-gene panel Mouse Fatty Liver qPCR array or (**b**) single qPCR (*n*=5–6 per group). (**a**) Values are average fold change of significant differences (*t*-test, *n*=4–5 per group, *P*<0.05) between diet within each genotype (diet effect) and between genotype within each dietary group (genotype effect) with green highlighting downregulation, red highlighting upregulation and grey highlighting indicating no significant difference. (**b**) Values are means±S.D. (bar graphs). Statistical significance indicated as *P<0.05

Differences in liver mass between HFD-fed mice were attributable to altered lipid accumulation. Macroscopically, *Casp2*^−/−^ mice were protected from HFD-induced NAFLD, whereas 10/16 HFD-fed WT mice had clear signs of hepatosteatosis (enlarged, white mottled liver), which was confirmed by histological analysis (Figure 2h). Analysis of liver gene expression using a fatty liver-specific quantitative PCR (qPCR) array revealed a small number (10/84) of significant differences between HFD-fed WT and *Casp2*^−/−^ mice (Figure 3a and Supplementary Data S1). These were mainly due to HFD-induced changes in WT but not *Casp2*^−/−^ mice as demonstrated by differences in the expression of the fatty acid transporter gene *CD36* (confirmed by individual qPCR) (Figure 3b). Importantly, no differences were observed in expression of any genes between SLD-fed WT and *Casp2*^−/−^ mice, including those involved in cholesterol and lipid metabolism/transport (Figure 3a and Supplementary Data S1).

Following 8 weeks of HFD feeding (13 weeks of age) glucose tolerance and insulin sensitivity were significantly improved in *Casp2*^−/−^ mice compared with WT controls as measured by intraperitoneal glucose tolerance testing (IPGTT) and insulin tolerance testing (IPITT) (Figures 2i and j). After 12 weeks (17 weeks of age), fasting blood glucose was significantly lower in both SLD- and HFD-fed *Casp2*^−/−^ mice (Figure 2k). This appeared to be independent of insulin as fasting plasma insulin levels did not differ between SLD-fed WT and *Casp2*^−/−^ mice (Figure 2k). In addition, *Casp2*^−/−^ mice did not develop HFD-induced hyperinsulinemia or insulin resistance as measured by the homeostasis model assessment of insulin resistance (HOMA-IR) value (Figure 2k).

Combined, these findings indicate that caspase-2 is involved in the maintenance of adipocyte size, function and glucose homeostasis and importantly that caspase-2 deficiency can improve adipose function and protects from HFD-induced obesity, NAFLD and fatty liver. Furthermore, the data indicate that the phenotype of HFD-fed *Casp2*^−/−^ mice is not the result of impaired lipid storage, rather there is a change in utilisation and/or metabolism of lipids in adipocytes.

### Shifts in whole-body fuel utilisation are maintained in HFD-fed *Casp2*^*−/−*^ mice

**Figure 4:**
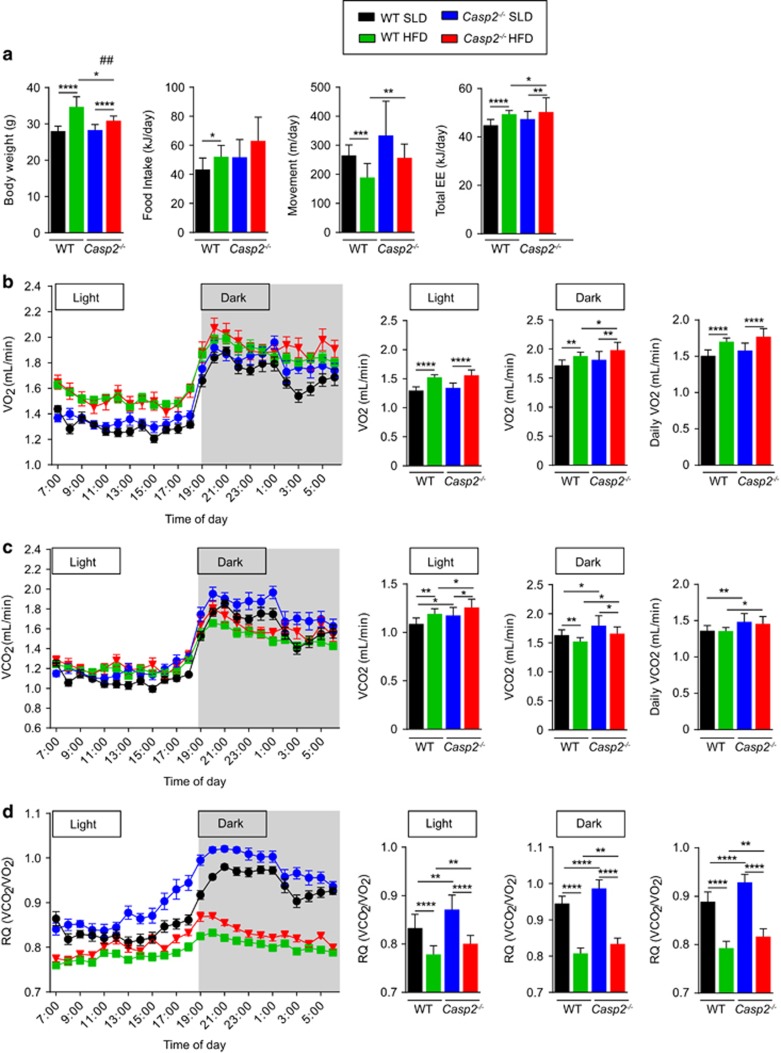
Increased carbohydrate utilisation is maintained in *Casp2*^−/−^ mice fed SLD or HFD. Metabolic monitoring with indirect calorimetry was performed on 13-week old WT and *Casp2*^−/−^ mice after 8 weeks of *ad libitum* SLD- or HFD- feeding. (**a**) Body weight, total daily food intake, activity (movement) and energy expenditure (EE). (**b**) VO_2_ consumption (**c**) VCO_2_ production and (**d**) RQ determined over 24-h period with 12-h light–dark cycles. Values are means±S.D. (bar graphs) and means±S.E.M (scatter plots) (*n*=10–12 per group). Pair-wise comparisons were made between diet within each genotype (diet effect) and between genotype within each dietary group (genotype effect). Statistical significance indicated as *P<0.05, **P<0.01, ***P<0.001, ****P<0.0001 and ^#^*P*<0.05 indicates effect of diet was different between genotypes as assessed by two-way ANOVA

After 8 weeks of HFD feeding (13 weeks of age), metabolic phenotyping was repeated. SLD-fed mice maintained the same metabolic phenotype as observed at 4 weeks of age (Figure 4 and Supplementary Figure S2). Food intake and feeding behaviour remained similar between genotypes on HFD (Figure 4a and Supplementary Figure S2) and this was despite differences in leptin levels (Figure 2e). Interestingly, HFD-fed *Casp2*^−/−^ mice had a small but significant increase in total daily energy expenditure corresponding with a similar increase in total daily movement (Figure 4a and Supplementary Figure S2). Fat oxidation requires increased oxygen consumption and HFD feeding is known to shift the balance in fuel choice towards increased fat utilisation.^20^ Consistent with this, in both genotypes, HFD feeding significantly increased total VO_2_ and decreased the RQ value (Figures 4b and d). However, HFD-fed *Casp2*^−/−^ mice maintained significantly higher levels of VCO_2_ and the RQ value when compared with HFD-fed WT controls (Figures 4c and d). Importantly, the magnitude of change in RQ value upon HFD feeding was similar in both genotypes. These data indicate that while *Casp2*^−/−^ mice maintain their preference for utilising carbohydrates as a fuel source they are not impaired in their ability to switch to utilising fats.

### Altered liver and skeletal muscle metabolism do not affect fuel choice in *Casp2*^*−/−*^ mice

**Figure 5:**
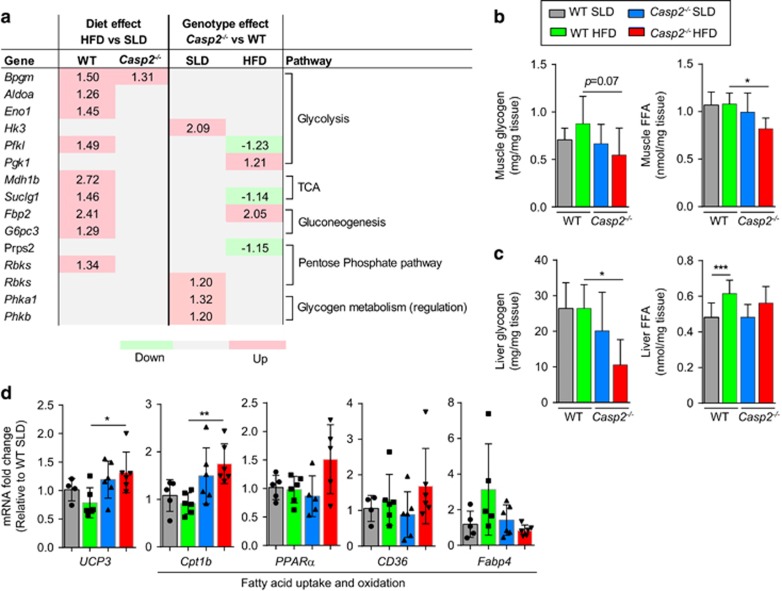
Gene expression analysis of skeletal muscle tissue from SLD- and HFD-fed WT and *Casp2*^−/−^ mice. (**a**) Expression of genes involved in glucose/glycogen metabolism identified as being significantly altered by diet or genotype as measured by an 84-gene panel Glucose Metabolism qPCR array. Values are average fold change of significant differences (*t*-test, *n*=3–4 per group, *P*<0.05) between diet within each genotype (diet effect) and between genotype within each dietary group (genotype effect) with green highlighting downregulation, red highlighting upregulation and grey highlighting indicating no significant difference. Glycogen and FFA levels were measured in (**b**) skeletal muscle (quadriceps; *n*=6 per group) and (**c**) liver (*n*=6 per group) from WT and *Casp2*^−/−^ mice fed SLD or HFD for 12 weeks. (**d**) qPCR used to measure expression of genes involved in fatty acid uptake and oxidation (*n*=5–6 per group). Values are means±S.D. Statistical significance indicated as *P<0.05, **P<0.01, ***P<0.001

Previously, we found activity of the mitochondrial oxidative phosphorylation (OXPHOS) complex III to be reduced in livers of *Casp2*^−/−^ mice.^9^ As inhibition of OXPHOS is known to result in increased glycolysis, this could account for the observed adaptive shift towards increased glycolytic (carbohydrate) fuel use in *Casp2*^−/−^mice.^21^ However, there was no significant difference in gene expression of key enzymes involved in glycolysis and fatty acid oxidation in livers of *Casp2*^−/−^ mice consistent with our previous findings.^8^ According to the Randle cycle, on the basis of substrate availability, increased fatty acid oxidation reduces glucose utilisation;^22^ conversely, impaired and reduced fatty acid oxidation can lead to increases in glucose utilisation. Increases in whole-body carbohydrate utilisation and protection from obesity can also been result from impaired mitochondrial fatty acid oxidation in skeletal muscle.^23^ Therefore, we carried out a gene expression screen of skeletal muscle (quadriceps) using a qPCR array specific for glucose/glycogen metabolism genes and found minimal differences between genotypes on both diets (Figure 5a and Supplementary Data S2). In addition, expression of key genes involved in fatty acid transport and oxidation did not differ between SLD-fed mice (Figure 5d). However, in *Casp2*^−/−^mice, HFD feeding significantly increased expression of mitochondria fatty acid transporter *Cpt1b* and the mitochondria uncoupling protein *UCP3* compared with SLD controls, as well as reduced levels of free fatty acids (FFAs) in skeletal muscle, but not liver (Figures 5b and d). In HFD-fed *Casp2*^−/−^ mice, glycogen levels were lower in both skeletal muscle and liver compared with WT controls but no differences were observed between SLD-fed mice (Figures 5b and c). These data indicate that altered mitochondrial function and/or metabolism in the liver and skeletal muscle of *Casp2*^−/−^mice are unlikely to have a major contributing role to the shift in whole-body fuel utilisation. In addition, this suggests that the tissue-specific responses to metabolism in HFD feeding may differ in *Casp2*^−/−^ mice.

### *Casp2*^*−/−*^ mice do not show changes in gene expression associated with insulin resistance in gWAT

**Figure 6:**
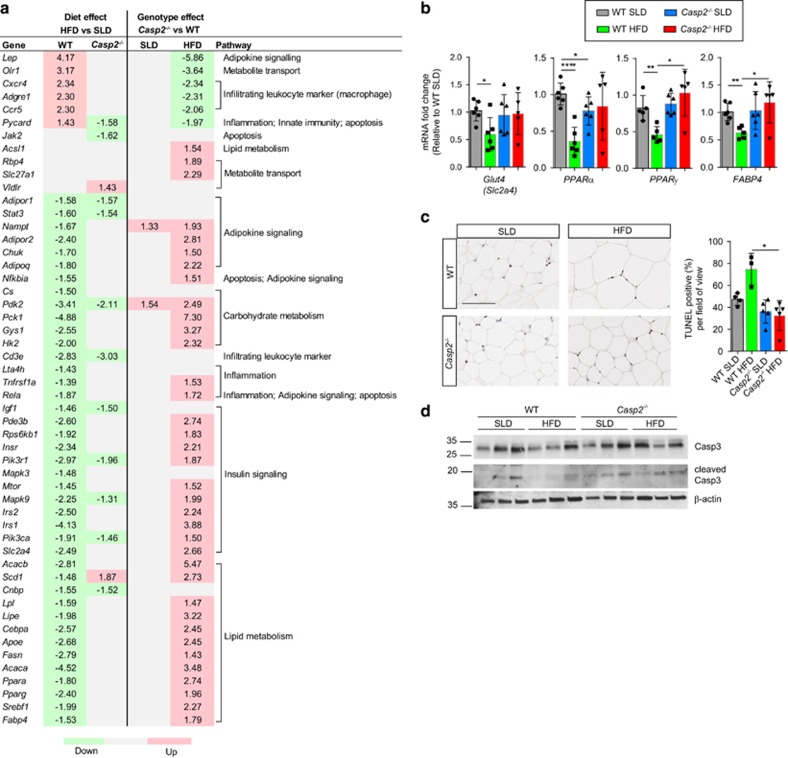
Expression analysis of insulin resistance genes in gWAT and adipocyte apoptosis in HFD-fed *Casp2*^−/−^ mice. Expression of genes known to be involved in insulin resistance identified as being significantly altered (fold change relative to SLD-fed WT mice>1.2 and *P*<0.05) in gonadal WAT (gWAT) by diet or genotype as measured by (**a**) an 84-gene panel Insulin Resistance qPCR array and verified by (**b**) individual qPCR analysis (*n*=5–6 per group). (**a**) Values are average fold change of significant differences (*t*-test, *n*=4–5 samples per group, *P*<0.05) between diet within each genotype (diet effect) and between genotype within each dietary group (genotype effect) with green highlighting downregulation, red highlighting upregulation and grey highlighting indicating no significant difference. (**c**) Representative images of TUNEL-positive cells in gWAT after 12 weeks of SLD- or HFD feeding. Bar graph displays % of TUNEL-positive cells per field of view. (*n*=3–5 per group). (**d**) Immunoblot analysis of total caspase-3 (Casp3) and cleaved Casp3 in iBAT tissue with *β*-actin as loading control. Values are means±S.D. Statistical significance indicated as *P<0.05, **P<0.01, ****P<0.0001

As adipocyte cell size positively correlates with glucose intolerance and hyperinsulinaemia in obesity,^24^ we screened gWAT gene expression against a panel of genes involved in insulin resistance. HFD feeding altered the expression of a large number of genes in WT but not *Casp2*^−/−^ mice, which resulted in a large number of significant differences between genotypes on HFD (Figure 6a and Supplementary Data S3). In WT mice, HFD decreased expression of 41/84 genes, many involved in glucose uptake, lipid metabolism insulin signalling and increased expression of 6/84 genes, whereas in *Casp2*^−/−^ mice, HFD altered expression of only 13/84 genes analysed (Figure 6a). The only significant difference observed between SLD-fed mice was an increase in expression of *Nampt* and *Pdk2* in *Casp2*^−/−^ mice compared with WT controls (Figure 6a).

In obesity, adipogenesis can be impaired by dysregulation of adipogenic genes.^25^ Indeed, several adipogenic genes, including *PPARγ* and *FABP4*, were decreased in HFD-fed WT but not *Casp2*^−/−^ mice (Figure 6a). These differences were confirmed by qPCR analysis as were differences in *Glut4* and *PPARα* expression (Figure 6b). Among the upregulated genes in HFD-fed WT mice were markers of infiltrating macrophages *Cxcr4*, *Adgre1* and *Ccr5* (Figure 6a).

### *Casp2*^*−/−*^ mice have reduced HFD-induced white adipocyte cell death

In obesity, inflammatory infiltration occurs in response to increases in apoptotic cell death of adipocytes, and is a key driver in the pathogenesis of obesity.^26^ Assessment of apoptosis by TUNEL revealed almost a two-fold increase in the number of TUNEL-positive dying white adipocytes in gWAT of HFD-fed WT mice compared with SLD-fed WT controls and HFD-fed *Casp2*^−/−^ mice (Figure 6c). Importantly, there was no difference in adipocyte cell death between SLD-fed WT and *Casp2*^−/−^ mice indicating the likely importance of a non-apoptotic role of caspase-2 in adipocyte biology.

Brown adipocyte cell death also contributes to the pathogenesis of obesity by decreasing thermogenic activity of BAT; however, no differences were observed between genotype or diets (Supplementary Figure 3a). Immunoblot analysis revealed an apparent decrease in pro-caspase-3 in gWAT and iBAT of HFD-fed WT mice but no difference in cleaved caspase-3 was detected (Figure 6d and Supplementary Figure 3b). Caspase-2 has been linked to lipoapoptosis^27^ and has been suggested to be involved in cell death of adipocytes following Western diet feeding.^16^ However, we observed no difference in protein levels of full-length caspase-2 or detection of its cleavage products in gWAT and iBAT of HFD-fed WT mice (data not shown).

### Caspase-2 deficiency increases HFD-induced browning of gWAT

**Figure 7:**
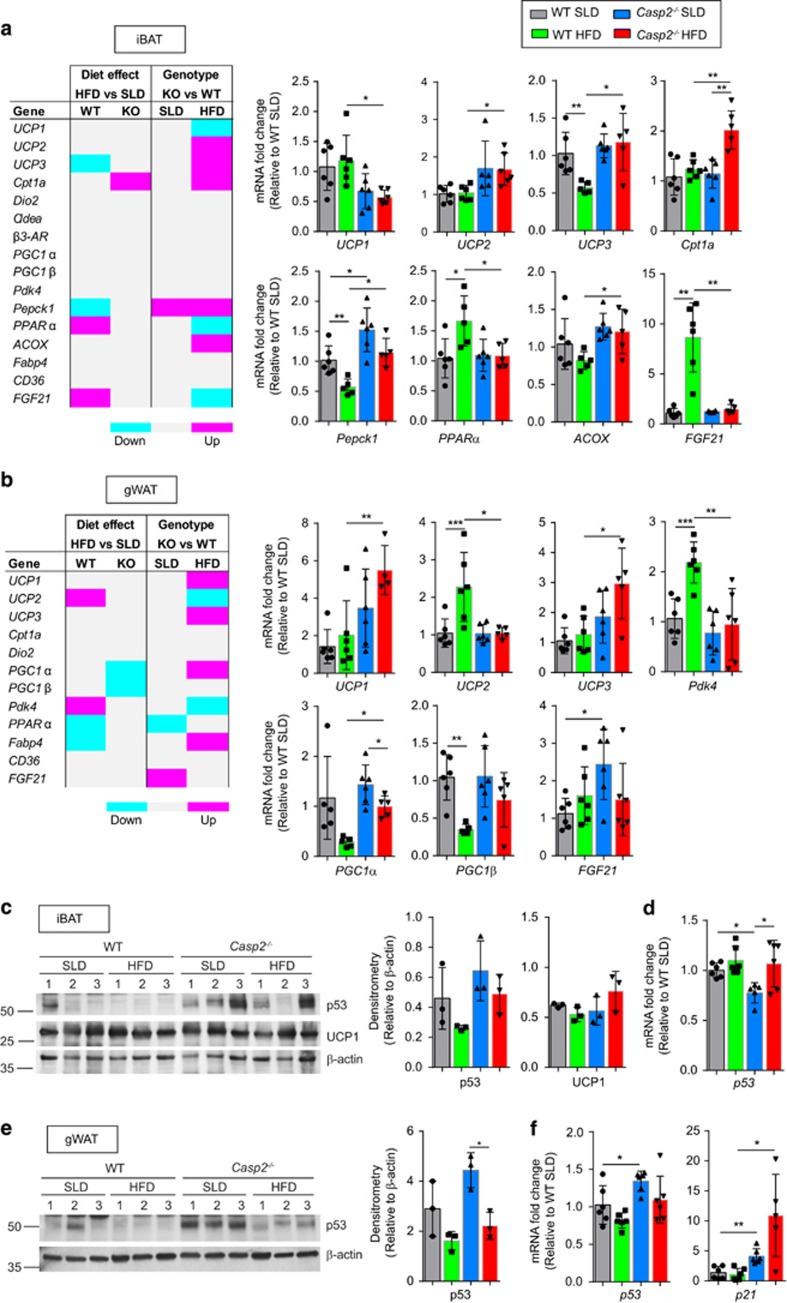
Caspase-2 deficiency increases *UCP1*levels in gWAT following HFD feeding and alters p53 expression. (**a** and **b**) Gene expression (relative to SLD-fed WT) measured relative by qPCR in (**a**) iBAT and (**b**) gWAT of mice fed SLD or HFD for 12 weeks. Heatmaps summarise genes significantly increased or decreased by diet or genotype. Bar graphs of expression for some selected genes are shown (*n*=5–6 per group). (**c-f**) Immunoblot analysis of p53 and UCP1 protein and gene expression of *p53* and *p21* in (**c** and **d**) iBAT and (**e** and **f**) gWAT of WT and *Casp2*^−/−^ mice fed HFD for 12 weeks with bar graphs in **c** and **d** displaying densitrometric analysis. *β*-Actin used as a loading control for immunoblots. Values are means±S.D. Statistical significance indicated as *P<0.05, **P<0.01, ***P<0.001

Adaptive thermogenesis in response to HFD feeding can occur as a means to try and mitigate the effects of increased lipid accumulation. To assess if this was altered by caspase-2, gene expression analysis was used to investigate differences in BAT thermogenesis and HFD-induced browning of WAT (e.g. recruitment/development of beige/brite cells). In iBAT, no differences were observed in the expression of thermogenic genes (such as *Cidea* and *PGC-1α*). Apart from significantly higher levels of *Pepck1*, there were also no differences in expression of genes involved in glucose and fatty acid transport/metabolism in SLD-fed *Casp2*^−/−^ mice (Figure 7a). Interestingly, levels of uncoupling protein 1 (*UCP1*), a marker of BAT/thermogenesis, were significantly lower in iBAT of HFD-fed *Casp2*^−/−^ mice compared with WT controls (Figure 7a) but UCP1 protein levels did not notably differ between genotype or diet (Figure 7c). In *Casp2*^−/−^mice, HFD feeding led to significantly increased expression of *Cpt1a* relative to SLD-fed mice, whereas in WT mice, significantly altered gene expression of *UCP2, Pepck1, PPARα* and *FGF*21 was observed (Figure 7a). In HFD-fed *Casp2*^−/−^ mice, the expression of *Cpt1a, UCP2, UCP3, Pepck1* and *ACOX* was significantly higher than observed in HFD-fed WT mice, whereas *PPARα* and *FGF21* were significantly lower (Figure 7a). This suggests that iBAT activity is not altered by caspase-2 deficiency and does not contribute to the shift in fuel choice or protection from obesity.

In gWAT, levels of *UCP1* were significantly higher in HFD-fed *Casp2*^−/−^ mice indicating a potential increase in browning (Figure 7b); however, UCP1 protein was not detectable by immunoblotting (data not shown). Levels of *UCP3* were also significantly higher in HFD-fed *Casp2*^−/−^ mice, whereas levels of *UCP2* were significantly lower (Figure 7c). Additional markers of browning, including *Cidea* and *β3*-AR were not detectable in gWAT and there was no difference in *Dio2* expression (Figure 7b). In contrast to iBAT, HFD feeding did not alter levels of *Cpt1a* in gWAT of *Casp2*^−/−^ mice (Figure 7b).

Adipose tissue p53 has been shown to have important roles in insulin resistance and thermogenesis.^28, 29^ As caspase-2 has also been linked with altered p53 response in several studies,^11, 30, 31, 32^ we investigated p53 expression in our samples. In both dietary groups, we observed increased levels of p53 protein in iBAT and gWAT of *Casp2*^−/−^ mice (Figures 7c and d) and a decrease in adipose p53 protein upon HFD feeding that was most notable in gWAT of *Casp2*^−/−^ mice (Figures 7c and d). This is opposite to the common observation of increased p53 following HFD feeding,^28, 33^ and may be attributable to the 6-h fast before tissue harvest although this was not confirmed. At the transcript level, we also observed higher levels of *p53* in gWAT of *Casp2*^−/−^ mice compared with WT under both SLD and HFD conditions, which corresponded with a significant increase in *p21* levels (Figure 7d). In contrast, we detected decreased *p53* levels in iBAT, in SLD-fed *Casp2*^−/−^ mice compared with WT controls (Figure 7c) and were unable to detect *p21* transcript in this tissue. These data indicate that p53 levels in iBAT are not associated and do not contribute to the observed protection from diet-induced obesity in *Casp2*^−/−^ mice.^26^

## Discussion

Caspase-2 has previously been implicated in lipid metabolism, glucose homeostasis and ageing.^8, 9, 11^ In this study, we show that altered glucose homeostasis in *Casp2*^−/−^ mice is the result of a whole-body shift in fuel choice towards increased carbohydrate utilisation. We show that caspase-2 is an intrinsic mediator of basal energy metabolism and provide further evidence to support a direct role for caspase-2 in adipocyte biology and fat expansion. In addition, we show that caspase-2 deficiency protects from the development of HFD-induced obesity, insulin resistance and NAFLD. A growing body of evidence now suggests that hyperinsulinemia precedes the development of insulin resistance^34^ and as such, altered insulin levels on HFD may be part of the mechanism of caspase-2 function. Similar to findings from Western diet fed mice,^16^ protection from obesity likely involves increased browning of WAT and reduced adipocyte cell death. However, as we have identified an intrinsic difference in fuel utilisation in the *Casp2*^−/−^ *mice* with no difference in adipocyte cell death under normal dietary conditions, it is unlikely that the apoptotic function of caspase-2 is a contributing factor to the metabolic function although this requires further investigation.

Altered substrate utilisation in *Casp2*^−/−^ mice is identifiable by the shift in RQ value. Although the change in RQ, although highly significant, appears to be small it is physiologically important as it is not because of a short-term change, for example, a short burst of exercise, but is a more sustained effect, already present immediately post-weaning of *Casp2*^−/−^ mice, which is likely to have long-term physiological outcomes. In addition, it is well established that RQ values will fall in adaptation to exposure to a HFD and this magnitude in humans has been observed to be in the range of 0.03,^35^ which is less than the observed difference in the *Casp2*^−/−^ mice.

Smaller adipocytes are known to be more insulin sensitive and thus have greater glucose uptake.^36^ Although altered glucose homeostasis in *Casp2*^−/−^ mice appears to be independent of insulin sensitivity, it is possible that the whole-body shift towards increased carbohydrate utilisation in *Casp2*^−/−^ mice is a direct result of the smaller adipocyte size and reduced adipose mass. Alternatively, altered metabolic flux in non-adipose tissues, such as skeletal muscle or liver, may result in increased energy demand and reliance on glycolysis. In turn, this may cause an adaptive response in WAT and be driving the phenotype of *Casp2*^−/−^ mice. However, we observed no major differences in key glucose or lipid metabolism genes in SLD-fed *Casp2*^−/−^ mice consistent with our previous findings.^8^ Interestingly, however, the HFD did upregulate expression of *UCP3* and *Cpt1a/b* in iBAT and skeletal muscle in *Casp2*^−/−^ mice. Although this suggests that an increase in fatty acid oxidation in these tissues, UCP3 has been identified as an important regulator of adaptive thermogenesis (increased heat production) in both skeletal muscle and BAT.^37^ Thus, this could be part of the mechanism providing protection from HFD-induced obesity and future studies will need to investigate if heat production is altered in *Casp2*^−/−^ mice.

An increase in *UCP1* expression in gWAT is indicative of increased browning of gWAT in HFD-fed *Casp2*^−/−^ mice and is similar to findings in Western diet fed *Casp2*^−/−^ mice.^16^ As browning of WAT is mostly due to *de novo* generation of new beige/brite adipocytes as opposed to recruitment or conversion of cells,^38^ the data suggest that hyperplasia may be increased in HFD-fed *Casp2*^−/−^ mice as a means to help mitigate the effects of excess lipid consumption. This is supported by the maintenance of *adiponectin* and *PPARγ* in HFD-fed *Casp2*^−/−^ mice in this study and the observation of increased proliferation of *Casp2*^−/−^ adipose-derived mesenchymal stem cells^16^ although differentiation potential was not assessed in that study. A decrease in bone marrow *PPARγ* in *Casp2*^−/−^ suggests that caspase-2 expression may be involved in adipocyte differentiation.^19^ However, we observed no difference in *PPARγ* gene expression in gWAT of SLD-fed *Casp2*^−/−^ mice in this study and previously observed no difference in *PPARγ* in aged fed and fasted *Casp2*^−/−^ livers or gWAT.^8^

Caspase-2 deficiency results in increased susceptibility to oxidative stress-induced damage and a mild-premature ageing phenotype.^11, 15^ In addition, glucose metabolism is generally upregulated in response to increase energy demand. Therefore, we propose that global deletion of caspase-2 alters metabolic pathways resulting in mild energy stress. In turn, this increases energy demand resulting in adaptive remodelling that drives the phenotype of *Casp2*^−/−^ mice. This would explain why caspase-2 deficiency results in favourable glucose homeostasis and protection from HFD-induced obesity while paradoxically increasing their susceptibility to oxidative stress-induced damage and premature ageing. Further studies will now be required to determine the tissue-specific roles of caspase-2 to help elucidate its precise molecular mechanism.

In conclusion, we have shown that caspase-2 is an intrinsic mediator of basal energy metabolism. In addition, we provide further evidence to support a direct role for caspase-2 in adipocyte biology, fat expansion and its potential as a therapeutic target from the treatment of obesity and metabolic disease. Although our findings demonstrate that the mechanism of caspase-2 in metabolism likely involves non-apoptotic functions, important questions remain as to what the substrates of caspase-2 are and whether the metabolic function is fully independent of the function of caspase-2 in growth arrest and apoptosis of cells carrying mitotic aberrations.^32, 39^

## Materials and methods

### Animal studies

Male WT and *Casp2*^−/−^ mice on a C57BL/6J background^11^ were used for experimental studies from 4-week of age. Animal ethics approval for this research was obtained from the South Australian Health and Medical Research Institute (SAHMRI) and University of Adelaide Animal Ethics Committees Animal Ethics Committees, in accordance with National Health and Medical Research Council of Australia guidelines. Mice were housed in pathogen-free conditions at the SAHMRI Bioresources Facility (SAHMRI, Adelaide, SA, Australia) under a 12-h light–dark cycle (lights on at 0600 hours) with constant temperature (20–23 °C) and *ad libitum* access to food and water. Mice were fed either SLD (Teklad global 18% protein rodent, irradiated diet #2918; Harlan, Indianapolis, IN, USA) or HFD (60% kJ from fat; made in-house as per Research Diets formula for D12492) for 12 weeks beginning at 5 weeks of age. Mice were killed at the same time of day in the light phase (1200–1400 hours) after a 6- to 8-h fast in order to stabilise systemic parameters and to allow the measurement of blood biochemistry in the fasting state. All animals were anaesthetised with isoflurane, blood collected by cardiac puncture and killed by cervical dislocation. Blood samples were collected in EDTA tubes for isolation of plasma. Tissues were collected upon killing, weighed (liver, gWAT and iBAT), snap-frozen in liquid nitrogen and maintained at −70 °C until analysed.

### Metabolic phenotyping

Metabolic measurements (food intake, locomotor activity, VO_2_ consumption and VCO_2_ production) were obtained using the Promethion metabolic phenotyping system (Sable Systems, Las Vegas, NV, USA). Mice were housed in the Promethion system for 48–72 h with *ad libitum* access to food and water. Monitoring was performed for 24–48 h after mice acclimatised to cages for 6–12 h. The RQ was calculated as the ratio of VCO_2_/VO_2_ with an RQ=0.7 indicative of pure fat oxidation and an RQ=1.0 indicative of pure carbohydrate oxidation.

### Tissue and serum biochemistry

Plasma triglycerides were measured by automated analysis (CSIRO, Adelaide, SA, Australia). Insulin and leptin plasma levels were measured using the Rat/Mouse insulin ELISA kit (cat # EZRMI-13 K) and Mouse Leptin ELISA kit (cat #EXML-82 K; Millipore, Bedford, MA, USA). Glycogen and FFAs in skeletal muscle and liver tissue were determined using commercially available assay kits (BioVision, Milpitas, CA, USA; Sigma, St. Louis, MO, USA).

### IPGTT, IPITT and insulin resistance

Glucose and insulin tolerance testing were performed following intraperitoneal injection of 1 g/kg glucose or 0.5 U/kg insulin/kg, respectively, after a 6- to 8-h fast as previously described.^8^ The HOMA-IR (HOMA-IR=fasting plasma insulin × fasting blood glucose/22.5) reflecting insulin resistance was calculated from fasting blood glucose and insulin levels measured after 12 weeks of HFD feeding using iHOMA2 Version 8.82.R2.^40^

### MRI body composition analysis

Whole-body fat and lean mass were determined via an 3D T_1_-weighted gradient echo acquired with a benchtop MRI system (Bruker Icon 1T; Bruker Physik GmbH, Ettlingen, Baden Wuerttemberg, Germany) in live, 17-week old mice, anaesthetised with isoflurane. ITK snap (www.itksnap.org) was used for consequent segmentation of the scans.

### Real-time qPCR

For standard qPCR, total RNA was extracted from frozen tissue, reverse transcribed and performed as previous described.^9^ Gene expression was normalised to housekeeping gene (*β*-actin or TATA-binding protein) and then expressed as fold change of SLD-fed WT mice using the 2^-ΔΔCT^ method. Primer sequences are provided in Supplementary Table S1.

For RT^2^ Profiler PCR array (Qiagen/SA Biosciences, Valencia, CA, USA) analysis, first-strand cDNA was synthesised from total RNA using the RT^2^ First strand cDNA synthesis kit (Qiagen). The RT^2^ Profiler PCR array system was set up, run and analysed on the Viaa 7 Real-Time PCR System (Thermo Fisher Scientific Inc., Wilmington, DE, USA) according to the manufacturer's instructions. RT^2^ Profiler PCR arrays used were: mouse fatty liver (#PAMM-157Z); mouse glucose metabolism (#PAMM-006Z); mouse insulin resistance (#PAMM-156Z, Qiagen/SA Biosciences). Gene lists and functional grouping are provided in Supplementary Data S1-S3. PCR arrays were 96 × 4 format allowing for four-independent samples to be run on each array. Data were processed using RT^2^ Profiler PCR Array Data Analysis version 3.5 online software (http://pcrdataanalysis.sabiosciences.com/pcr/arrayanalysis.php).

### Immunoblotting

Proteins were isolated from tissues, resolved by SDS-PAGE then transferred to PVDF membrane as previously described.^8^ Membranes were immunoblotted with the primary antibodies specific to: caspase-3 (clone 8G10, #9665), cleaved caspase-3 (#9664) and p53 (clone 1C12, #2524; Cell Signalling Technology, Beverly, MA, USA); UCP1 (#ab10983; Abcam, Cambridge, MA, USA); and *β*-actin clone AC-15 (#A5441; Sigma-Aldrich, St. Louis, MO, USA). Densitrometric analysis was performed using Image J software (NIH, Bethesda, MD, USA).

### Histology and TUNEL

Standard methods were used for haematoxylin/eosin staining of tissues fixed in HistoChoice (Sigma-Aldrich). TUNEL staining and quantitation of apoptotic cells was performed as described.^13^

### Statistical analysis

Statistical analysis was performed using GraphPad Prism software (v 6.0, San Diego, CA, USA). Data are expressed as mean±S.D. or mean±S.E.M. For pair-wise comparisons a two-tailed unpaired *t-*test with Welch’s corrections was used. For multi-group comparisons, one-way or two-way ANOVA was used with Tukey’s *post hoc* testing unless stated otherwise. Values of *P*<0.05 were considered statistically significant

## References

[bib1] Hardie DG. Organismal carbohydrate and lipid homeostasis. Cold Spring Harb Perspect Biol 2012; 4. 10.1101/cshperspect.a006031 PMC3331699 22550228

[bib2] Riera CE, Dillin A. Tipping the metabolic scales towards increased longevity in mammals. Nat Cell Biol 2015; 17: 196–203. 25720959 10.1038/ncb3107

[bib3] Hanahan D, Weinberg RA. Hallmarks of cancer: the next generation. Cell 2011; 144: 646–674. 21376230 10.1016/j.cell.2011.02.013

[bib4] Lopez-Otin C, Blasco MA, Partridge L, Serrano M, Kroemer G. The hallmarks of aging. Cell 2013; 153: 1194–1217. 23746838 10.1016/j.cell.2013.05.039 PMC3836174

[bib5] Stanley IA, Ribeiro SM, Gimenez-Cassina A, Norberg E, Danial NN. Changing appetites: the adaptive advantages of fuel choice. Trends Cell Biol 2014; 24: 118–127. 24018218 10.1016/j.tcb.2013.07.010 PMC3946676

[bib6] Miles MA, Kitevska-Ilioski T, Hawkins CJ. Old and novel functions of caspase-2. Int Rev Cell Mol Biol 2017; 332: 155–212. 28526132 10.1016/bs.ircmb.2016.12.002

[bib7] Puccini J, Dorstyn L, Kumar S. Caspase-2 as a tumour suppressor. Cell Death Differ 20: 1133–11339. 10.1038/cdd.2013.87 PMC3741511 23811850

[bib8] Wilson CH, Nikolic A, Kentish SJ, Shalini S, Hatzinikolas G, Page AJ et al. Sex-specific alterations in glucose homeostasis and metabolic parameters during ageing of caspase-2-deficient mice. Cell Death Disco 2016; 2: 16009. 10.1038/cddiscovery.2016.9 PMC4979492 27551503

[bib9] Wilson CH, Shalini S, Filipovska A, Richman TR, Davies S, Martin SD et al. Age-related proteostasis and metabolic alterations in Caspase-2-deficient mice. Cell Death Dis 2015; 6: e1597. 25611376 10.1038/cddis.2014.567 PMC4669765

[bib10] Zhang Y, Padalecki SS, Chaudhuri AR, De Waal E, Goins BA, Grubbs B et al. Caspase-2 deficiency enhances aging-related traits in mice. Mech Ageing Dev 2007; 128: 213–221. 17188333 10.1016/j.mad.2006.11.030 PMC1828128

[bib11] Shalini S, Dorstyn L, Wilson C, Puccini J, Ho L, Kumar S. Impaired antioxidant defence and accumulation of oxidative stress in caspase-2-deficient mice. Cell Death Differ 2012; 19: 1370–1380. 22343713 10.1038/cdd.2012.13 PMC3392626

[bib12] Ho LH, Taylor R, Dorstyn L, Cakouros D, Bouillet P, Kumar S. A tumor suppressor function for caspase-2. Proc Natl Acad Sci USA 2009; 106: 5336–5341. 19279217 10.1073/pnas.0811928106 PMC2664004

[bib13] Shalini S, Nikolic A, Wilson CH, Puccini J, Sladojevic N, Finnie J et al. Caspase-2 deficiency accelerates chemically induced liver cancer in mice. Cell Death Differ 2016; 23: 1727–1736. 27518436 10.1038/cdd.2016.81 PMC5041200

[bib14] Puccini J, Shalini S, Voss AK, Gatei M, Wilson CH, Hiwase DK et al. Loss of caspase-2 augments lymphomagenesis and enhances genomic instability in Atm-deficient mice. Proc Natl Acad Sci USA 2013; 110: 19920–19925. 24248351 10.1073/pnas.1311947110 PMC3856814

[bib15] Shalini S, Puccini J, Wilson CH, Finnie J, Dorstyn L, Kumar S. Caspase-2 protects against oxidative stress *in vivo*. Oncogene 2015; 34: 4995–5002. 25531319 10.1038/onc.2014.413

[bib16] Machado MV, Michelotti GA, Jewell ML, Pereira TA, Xie G, Premont RT et al. Caspase-2 promotes obesity, the metabolic syndrome and nonalcoholic fatty liver disease. Cell Death Dis 2016; 7: e2096. 26890135 10.1038/cddis.2016.19 PMC5399190

[bib17] Ferreira DM, Castro RE, Machado MV, Evangelista T, Silvestre A, Costa A et al. Apoptosis and insulin resistance in liver and peripheral tissues of morbidly obese patients is associated with different stages of non-alcoholic fatty liver disease. Diabetologia 2011; 54: 1788–1798. 21455726 10.1007/s00125-011-2130-8

[bib18] Machado MV, Michelotti GA, Pereira TD, Boursier J, Kruger L, Swiderska-Syn M et al. Reduced lipoapoptosis, hedgehog pathway activation and fibrosis in caspase-2 deficient mice with non-alcoholic steatohepatitis. Gut 2014; 64: 1148–1157. 25053716 10.1136/gutjnl-2014-307362 PMC4303564

[bib19] Coe LM, Lippner D, Perez GI, McCabe LR. Caspase-2 deficiency protects mice from diabetes-induced marrow adiposity. J Cell Biochem 2011; 112: 2403–2411. 21538476 10.1002/jcb.23163

[bib20] Scott CB. Contribution of anaerobic energy expenditure to whole body thermogenesis. Nutr Metab 2005; 2: 14. 10.1186/1743-7075-2-14 PMC1182393 15958171

[bib21] Marin-Buera L, Garcia-Bartolome A, Moran M, Lopez-Bernardo E, Cadenas S, Hidalgo B et al. Differential proteomic profiling unveils new molecular mechanisms associated with mitochondrial complex III deficiency. J Proteomics 2015; 113: 38–56. 25239759 10.1016/j.jprot.2014.09.007 PMC4259860

[bib22] Randle PJ, Garland PB, Hales CN, Newsholme EA. The glucose fatty-acid cycle. Its role in insulin sensitivity and the metabolic disturbances of diabetes mellitus. Lancet 1963; 1: 785–789. 13990765 10.1016/s0140-6736(63)91500-9

[bib23] Wicks SE, Vandanmagsar B, Haynie KR, Fuller SE, Warfel JD, Stephens JM et al. Impaired mitochondrial fat oxidation induces adaptive remodeling of muscle metabolism. Proc Natl Acad Sci USA 2015; 112: E3300–E3309. 26056297 10.1073/pnas.1418560112 PMC4485116

[bib24] Weyer C, Foley JE, Bogardus C, Tataranni PA, Pratley RE. Enlarged subcutaneous abdominal adipocyte size, but not obesity itself, predicts type II diabetes independent of insulin resistance. Diabetologia 2000; 43: 1498–1506. 11151758 10.1007/s001250051560

[bib25] White UA, Stephens JM. Transcriptional factors that promote formation of white adipose tissue. Mol Cell Endocrinol 2010; 318: 10–14. 19733624 10.1016/j.mce.2009.08.023 PMC3079373

[bib26] Alkhouri N, Gornicka A, Berk MP, Thapaliya S, Dixon LJ, Kashyap S et al. Adipocyte apoptosis, a link between obesity, insulin resistance, and hepatic steatosis. J Biol Chem 2010; 285: 3428–3438. 19940134 10.1074/jbc.M109.074252 PMC2823448

[bib27] Segear Johnson E, Lindblom KR, Robeson A, Stevens RD, Ilkayeva OR, Newgard CB et al. Metabolomic profiling reveals a role for caspase-2 in lipoapoptosis. J Biol Chem 2013; 288: 14463–14475. 23553630 10.1074/jbc.M112.437210 PMC3656301

[bib28] Minamino T, Orimo M, Shimizu I, Kunieda T, Yokoyama M, Ito T et al. A crucial role for adipose tissue p53 in the regulation of insulin resistance. Nature Med 2009; 15: 1082–1087. 19718037 10.1038/nm.2014

[bib29] Al-Massadi O, Porteiro B, Kuhlow D, Kohler M, Gonzalez-Rellan MJ, Garcia-Lavandeira M et al. Pharmacological and genetic manipulation of p53 in brown fat at adult but not embryonic stages regulates thermogenesis and body weight in male mice. Endocrinol 2016; 157: 2735–2749. 10.1210/en.2016-1209 27183316

[bib30] Dorstyn L, Puccini J, Wilson CH, Shalini S, Nicola M, Moore S et al. Caspase-2 deficiency promotes aberrant DNA-damage response and genetic instability. Cell Death Differ 2012; 19: 1288–1298. 22498700 10.1038/cdd.2012.36 PMC3392630

[bib31] Oliver TG, Meylan E, Chang GP, Xue W, Burke JR, Humpton TJ et al. Caspase-2-mediated cleavage of Mdm2 creates a p53-induced positive feedback loop. Mol Cell 2011; 43: 57–71. 21726810 10.1016/j.molcel.2011.06.012 PMC3160283

[bib32] Fava LL, Schuler F, Sladky V, Haschka MD, Soratroi C, Eiterer L et al. The PIDDosome activates p53 in response to supernumerary centrosomes. Genes Dev 2017; 31: 34–45. 28130345 10.1101/gad.289728.116 PMC5287111

[bib33] Tinahones FJ, Coin Araguez L, Murri M, Oliva Olivera W, Mayas Torres MD, Barbarroja N et al. Caspase induction and BCL2 inhibition in human adipose tissue: a potential relationship with insulin signaling alteration. Diabetes Care 2013; 36: 513–521. 23193206 10.2337/dc12-0194 PMC3579349

[bib34] Czech MP. Insulin action and resistance in obesity and type 2 diabetes. Nat Med 2017; 23: 804–814. 28697184 10.1038/nm.4350 PMC6048953

[bib35] Smith SR, Jonge LD, Zachwieja JJ, Roy H, Nguyen T, Rood JC et al. Fat and carbohydrate balances during adaptation to a high fat diet. Am J Clin Nutr 2000; 71: 450–457. 10648257 10.1093/ajcn/71.2.450

[bib36] Arner E, Westermark PO, Spalding KL, Britton T, Ryden M, Frisen J et al. Adipocyte turnover: relevance to human adipose tissue morphology. Diabetes 2010; 59: 105–109. 19846802 10.2337/db09-0942 PMC2797910

[bib37] Riley CL, Dao C, Kenaston MA, Muto L, Kohno S, Nowinski SM et al. The complementary and divergent roles of uncoupling proteins 1 and 3 in thermoregulation. J Physiol 2016; 594: 7455–7464. 27647490 10.1113/JP272971 PMC5157057

[bib38] Wang QA, Tao C, Gupta RK, Scherer PE. Tracking adipogenesis during white adipose tissue development, expansion and regeneration. Nat Med 2013; 19: 1338–1344. 23995282 10.1038/nm.3324 PMC4075943

[bib39] Dawar S, Lim Y, Puccini J, White M, Thomas P, Bouchier-Hayes L et al. Caspase-2-mediated cell death is required for deleting aneuploid cells. Oncogene 2017; 36: 2704–2714. 27991927 10.1038/onc.2016.423 PMC5442422

[bib40] Hill NR, Levy JC, Matthews DR. Expansion of the homeostasis model assessment of beta-cell function and insulin resistance to enable clinical trial outcome modeling through the interactive adjustment of physiology and treatment effects: iHOMA2. Diabetes Care 2013; 36: 2324–2330. 23564921 10.2337/dc12-0607 PMC3714535

