# Supplementary material for: Caspase-2 deficiency enhances whole-body carbohydrate utilisation and prevents high-fat diet-induced obesity
Source: Cell Death Dis. 2017 Oct 26;8(10):e3136–. doi: 10.1038/cddis.2017.518 (PMC5682682; doi:10.1038/cddis.2017.518)
Supplement: Supplementary Information [file cddis2017518x1.pdf]

SUPPLEMENTARY INFORMATION

Caspase-2 deficiency enhances whole-body carbohydrate utilisation and prevents high-fat diet induced obesity

Claire H Wilson[1], Andrej Nikolic[1], Stephen J Kentish[2,3], Marianne Keller[2,3] George Hatzinikolas[2], Loretta Dorstyn[1], Amanda J Page[2,3*], Sharad Kumar[1]*

[1]Centre for Cancer Biology, University of South Australia, Adelaide, SA 5001, Australia
[2]Adelaide Medical School, University of Adelaide, Adelaide, SA 5000, Australia
[3] South Australian Health and Medical Research Institute (SAHMRI), Adelaide, SA 5000, Australia

Supplementary Figure S1. Caspase-2 does not alter feeding behaviour in 4-week` old mice.
Metabolic monitoring with indirect calorimetry performed on 4-week old WT and *Casp2*$^{-/-}$ mice after 1 week of *ad libitum* SLD-feeding was used to determine the meal size, meal number, meal duration, intermeal duration and eating rate in mice throughout the (**a**) light and (**b**) dark cycles. Measurements were determined over a 24 h period with 12 h light-dark cycles. Values are means ± S.D. (bar graphs) and means ± S.E.M (scatter plots) (n=22-23/group). Statistical significance indicated as * $p < 0.05$, **$p < 0.01$, ***$p < 0.001$.

Supplementary Figure S2. Caspase-2 does not alter feeding behaviour in 13-week old mice after 8-weeks of SLD and HFD-fed mice
Metabolic monitoring with indirect calorimetry was performed on 13-week old WT and *Casp2*$^{-/-}$ mice after 8-weeks of *ad libitum* SLD- or HFD- feeding was used to determine the meal size, meal number, meal duration, intermeal duration and eating rate in mice throughout the (**a**) light and (**b**) dark cycles. Measurements were determined over 24 h period with 12 h light-dark cycles. Values are means ± S.D. (bar graphs) and means ± S.E.M (scatter plots) (n=10-12/group). Statistical significance indicated as * $p < 0.05$, **$p < 0.01$, ***$p < 0.001$, ****$p < 0.0001$.

Supplementary Figure S3. Caspase-2 does not alter brown adipocyte apoptosis following HFD feeding.
Brown adipocyte apoptosis was assessed in WT and *Casp2*$^{-/-}$ mice fed SLD or HFD for 12 weeks. (**b**) Representative images of TUNEL-positive cells in interscapular brown adipose tissue (iBAT) of mice after 12 weeks of SLD- or HFD-feeding. Bar graph displays % of TUNEL positive cells per field of view (n=3/group). (**b**) Immunoblot analysis of total caspase-3 (Casp3) and cleaved Casp3 in iBAT tissue with β-actin as loading control.

Supplementary Data S1-S3. Complete gene list and functional gene grouping for qPCR arrays. Information provided for the RT^2 Profiler™ PCR Arrays (**S1**) Mouse Fatty Liver, (**S2**) Mouse Glucose Metabolism and (**S3**) Mouse Insulin Resistance.

Supplementary Table S1. Sequences of primers used for qPCR analysis

**Gene**	**Forward primer (5'-3')**	**Reverse primer (5' -3')**
ACOX1	GCTGAGGAACCTGTGTCTCT	TCAAAGGCATCCACCAAAGC
Adiponectin	TGACGACACCAAAAGGGCTC	CACAAGTTCCCTTGGGTGGA
β-Actin	GATCATTGCTCCTCCTGAGC	AGTCCGCCTAGAAGCACTTG
β3-AR	CCTTCCGTCGTCTTCTGTGT	CCATCAAACCTGTTGAGCGG
CD36	AGATGACGTGGCAAAGAACAG	CCTTGGCTAGATAACGAACTCTG
Cidea	GCCGTGTTAAGGAATCTGCTG	TGCTCTTCTGTATCGCCCAGT
Cpt1a	CCTGGGCATGATTGCAAAG	GGACGCCACTCACGATGTT
Cpt1b	GCACACCAGGCAGTAGCTTT	CAGGAGTTGATTCCAGACAGGTA
Dio2	CAAACAGGTTAAACTGGGTGAA	GCACTGGCAAAGTCAAGAAGG
Fabp4	AAGGTGAAGAGCATCATAACCCT	TCACGCCTTTCATAACACATTCC
Fgf21	CTGGGGGTCTACCAAGCATA	CACCCAGGATTTGAATGACC
Slc2a4 (Glut4)	GTCCTCCTGCTTGGCTTCTT	AGCTGAGATCTGGTCAAACG
Leptin	CAGGATCAATGACATTTCACACA	GCTGGTGAGGACCTGTTGAT
p21	AGTGTGCCGTTGTCTCTTCG	ACACCAGAGTGCAAGACAGC
p53	CTCACTCCAGCTACCTGAAGA	AGAGGCAGTCAGTCAGTCTGA
Pdk4	AGAAGACCAGAAAGCCCTGTCA	GCCATTGTAGGGACCACATTATG
Pepck1	ATCATCTTTGGTGGCCGTAG	CATGGCTGCTCCTACAAACA
PGC1α	CGCCGTGTGATTTACGTTGG	GCTGTCTCCATCATCCCGC
PGC1β	TCTGACGTGGACGAGCTTTC	GTGCCATCCACCTTGACACA
PPARα	ATGCCAGTACTGCCGTTTTC	CCGAATCTTTCAGGTCGTGT
PPARγ	GTCACACTCTGACAGGAGCC	AGAACGTGACTTCTCAGCCC
UCP1	CTAGGGACCATCACCACCCT	GCAGGTGTTTCTCTCCCTGAA
UCP2	ATGGTTGGTTTCAAGGCCACA	CGGTATCCAGAGGGAAAGTGAT
UCP3	CAGCTTCCTCCCTGAACTGA	CAGGGGAAAAGTGAGGAGGT
