# Supplementary figures and images for: Caspase-2 deficiency enhances whole-body carbohydrate utilisation and prevents high-fat diet-induced obesity
Source: Cell Death Dis. 2017 Oct 26;8(10):e3136–. doi: 10.1038/cddis.2017.518 (PMC5682682; doi:10.1038/cddis.2017.518)

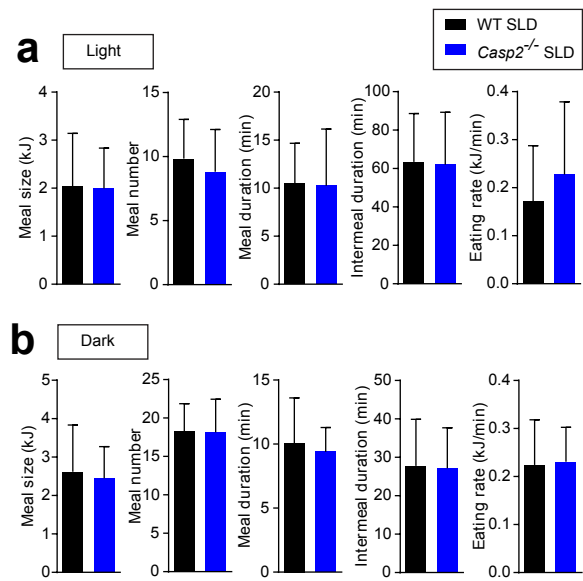

**Supplementary Figure S1**

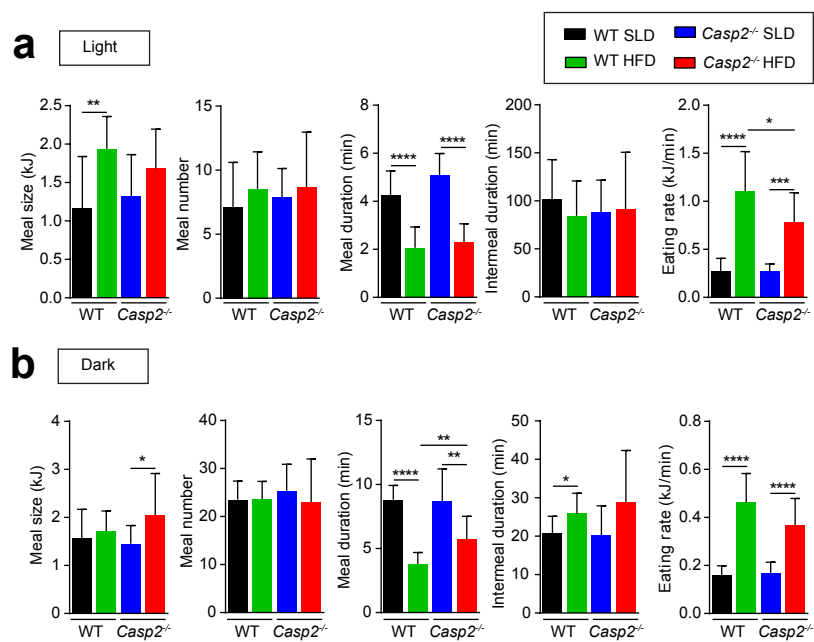

**Supplementary Figure S2**

**a**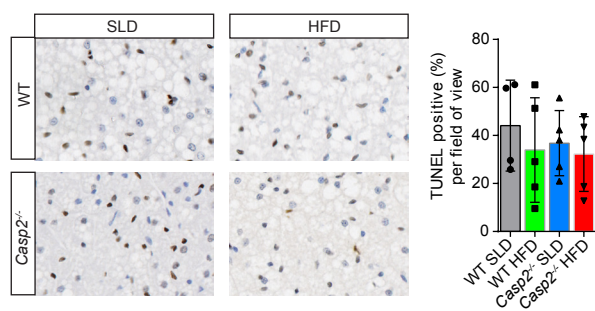**b**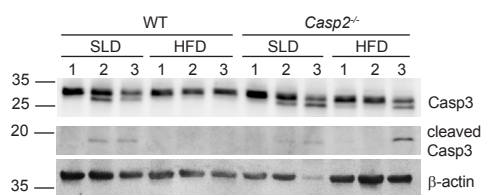**Supplementary Figure S3**

Supplement: Supplementary Figures [file cddis2017518x2.pdf]
